# Comparison of Magnetic Resonance Spectra Acquired With Hybrid PET/MR and Standalone MR Scanners

**DOI:** 10.1002/jmri.70056

**Published:** 2025-08-04

**Authors:** Carter B. Macdonald, Aditya Bhattacharya, Benjamin B. Risk, Taylor M. Zuleger, Sagar Mandava, Ralph Noeske, Candace C. Fleischer

**Affiliations:** ^1^ Department of Biomedical Engineering Georgia Institute of Technology and Emory University School of Medicine Atlanta Georgia USA; ^2^ Department of Neuroscience Georgia Institute of Technology Atlanta Georgia USA; ^3^ Department of Radiology and Imaging Sciences Emory University School of Medicine Atlanta Georgia USA; ^4^ Department of Biostatistics and Bioinformatics Rollins School of Public Health, Emory University Atlanta Georgia USA; ^5^ Emory Sports Performance and Research Center (SPARC) Flowery Branch Georgia USA; ^6^ Emory Sports Medicine Center Atlanta Georgia USA; ^7^ Department of Orthopaedics Emory University School of Medicine Atlanta Georgia USA; ^8^ GE HealthCare Waukesha Wisconsin USA

**Keywords:** brain, hybrid MR scanners, metabolite quantification, MR spectroscopy, PET/MR

## Abstract

**Background:**

The effect of the positron emission tomography (PET) modality in hybrid PET/MR scanners on MR spectral quality is unclear.

**Purpose:**

To evaluate spectral quality, quantification, and repeatability of brain MR spectroscopy (MRS) acquired on PET/MR and standalone MR scanners.

**Study Type:**

Prospective.

**Subjects:**

23 healthy adults (male/female = 11/12, age = 27 ± 11 years).

**Field Strength/Sequence:**

3T, semi‐localized by adiabatic selective refocusing.

**Assessment:**

Single voxel MR spectra were acquired with GE Signa PET/MR and Siemens Prisma Fit MR scanners in healthy volunteers, and additionally with a GE Premier MR scanner in vitro. Spectral quality, metabolite concentrations, and repeatability were evaluated.

**Statistical Tests:**

Linear mixed models assessed signal‐to‐noise ratio (SNR), full width at half maximum (FWHM), and metabolites/total creatine (tCr). Intraclass correlation coefficients (ICCs) determined repeatability; mean squared error (MSE) evaluated accuracy of quantification. Values are reported as mean ± standard deviation. Significance was determined by *p* ≤ 0.05.

**Results:**

In vivo spectra acquired using the Siemens standalone MR showed significantly higher SNR (PET/MR: 118.8 ± 19.1, MR: 132.2 ± 24.4) but significantly broader FWHM (PET/MR: 7.2 ± 1.8 Hz, MR: 8.4 ± 2.5 Hz) compared to PET/MR. All metabolite ratios were significantly higher in spectra acquired on the standalone MR. Within‐session repeatability was good (ICC > 0.75), and between‐session repeatability was moderate to excellent (ICC 0.5 to > 0.90) for all metabolites in vivo. In vitro spectra acquired with the PET/MR had significantly higher SNR (329.1 ± 46.1) than the Siemens standalone MR (150.8 ± 49.3) but significantly lower than the GE standalone MR (395.9 ± 33.7). The PET/MR produced narrower FWHM (2.1 ± 0.4 Hz) than the Siemens standalone MR (4.1 ± 1.7 Hz) but broader than the GE standalone MR (1.6 ± 0.1 Hz) and lower MSE for some metabolites.

**Data Conclusion:**

MR spectral quality appears uncompromised when acquired with hybrid PET/MR compared to standalone MR.

**Summary**

- Plain language summary
  ○ Many hospitals use “hybrid” imaging scanners, for example, a positron emission tomography (PET) and magnetic resonance imaging (MRI) combined into a single scanner.
  ○ Hybrid PET/MRI scanners reduce the time required for patients as PET and MRI scans are acquired at the same time. It is unclear, however, if combined scanners can produce the same data quality.
  ○ In healthy volunteers, we compared MRI data from a hybrid PET/MRI to data from a regular MRI.
  ○ We obtained high‐quality MRI data using a hybrid PET/MRI scanner. For patients who need to undergo both PET and MRI scans, hybrid scanners may be a viable option.

## 1 Introduction

Hybrid magnetic resonance (MR) scanners, such as positron emission tomography (PET)/MR, are increasingly common in clinical and research settings, enabling both MR and PET acquisition in a single comprehensive scan [1, 2, 3, 4, 5]. Clinical usage of PET/MR is largely focused on oncologic applications in brain, head and neck, and liver [6, 7]. Recent studies have also highlighted utility in breast and prostate imaging, where combined PET/MR may facilitate improved detection and monitoring of disease progression and response to therapy [8, 9, 10, 11]. In patients with high‐risk prostate cancer, the inclusion of PET/MR demonstrated the highest sensitivity for detection of metastatic sites and accurate disease staging, outperforming single modality imaging [4, 6, 9, 12]. Applications in brain imaging have also been demonstrated [13, 14], for example, the use of hybrid PET/MR compared to MR alone resulted in increased accuracy when differentiating between Alzheimer's disease and frontotemporal lobar degeneration [15].

An outstanding question regarding hybrid imaging scanners, including PET/MR, is the presence of the additional modality, that is, the PET detector, and its potential effects on the quality of MR data [16]. The integration of the PET detector can introduce inhomogeneities in the magnetic field, resulting in eddy currents and subsequent artifacts [17, 18, 19, 20, 21]. Recent studies have assessed PET and MR performance both independently and during simultaneous acquisition on fully hybrid PET/MR scanners as well as MR scanners using a PET insert [22, 23, 24, 25]. For example, a prior study using a standalone preclinical 7T MR scanner with a PET insert found non‐gradient‐intensive MR imaging (MRI) sequences were largely unaffected by the PET insert, whereas gradient‐intensive sequences showed a reduction in signal‐to‐noise ratio (SNR) by as much as 33% [26]. An increase in *B*_0_ field inhomogeneity was also observed and attributed to the presence of the PET insert, though *B*_1_ power was not affected [26]. While prior work has examined some effects of the PET detector on MR quality in hybrid PET/MR, quantitative evaluations of the impact on MRI or MR spectroscopy (MRS) data are largely absent.

A clear advantage of hybrid PET/MR scanners is the ability to obtain complementary metabolic information acquired from PET and MRS simultaneously [27, 28, 29, 30]. PET acquisition relies on a radioactive tracer, for example, ^18^F‐fluorodeoxyglucose (FDG), to assess metabolic activity and flux [31, 32]. While PET provides important metabolic and biochemical information, most applications rely on a single radioisotope. In comparison, MRS facilitates simultaneous quantification of a broad range of endogenous metabolites, enabling a more comprehensive profile of steady‐state metabolism [33, 34, 35, 36, 37]. Hybrid PET/MR scanners provide clear advantages for metabolic imaging, combining the benefits of MRS with the direct metabolic analysis provided by PET. MRS, however, is particularly sensitive to B_0_ and B_1_ field inhomogeneities, which can be introduced by the PET detector [17], potentially affecting spectral quality and metabolite quantification. The combined use of PET and MRS requires evaluation of the impact of the PET insert on MRS metrics.

The goal of this study was to systematically evaluate MR spectral quality, repeatability, and accuracy of metabolite quantification from spectra acquired using a hybrid PET/MR scanner compared to standalone MR scanners of the same field strength.

## 2 Materials and Methods

### 2.1 Study Participants

This prospective study was approved by the Emory University Institutional Review Board, and all participants provided written informed consent prior to participation. Inclusion criteria were participants who were medically healthy and at least 18 years of age. Exclusion criteria were < 18 years old, a positive pregnancy test for biological females, chronic or acute medical conditions, or contraindications to MR, including metal or implants not compatible with the 3T MR field strength and claustrophobia. Additionally, data was excluded if a participant exhibited excessive motion during the scan, identified by gross motion of the body and/or head during MRS acquisition combined with poor spectral quality observed in real time during acquisition (i.e., high noise, absence of clearly defined peaks in cumulative spectrum).

### 2.2 MR Data Acquisition

MR data were acquired using three 3T scanners: a GE Signa hybrid PET/MR (19‐channel head and neck radiofrequency (RF) receive coil, pre‐upgrade software version 26.003, post‐upgrade software version 30.1), a standalone Siemens MAGNETOM Prisma Fit MR (32‐channel head and neck receive coil, software version VE11C), and a standalone GE Signa Premier MR (21‐channel head and neck receive coil, software version 29.1) with similar capabilities (GE PET/MR: 44 mT/m gradient amplitude, 200 T/m/s slew rate, 60 cm bore; Siemens standalone MR scanner: 80 mT/m gradient amplitude, 200 T/m/s slew rate, 60 cm bore; GE standalone MR scanner: 80 mT/m gradient amplitude, 200 T/m/s slew rate, 70 cm bore). In vivo data was collected using the hybrid GE PET/MR and Siemens standalone MR scanners, while in vitro data was collected using all three scanners (GE PET/MR, Siemens standalone MR, and GE standalone MR). Sequence parameters were matched across scanners.

A *T*_1_‐weighted magnetization‐prepared rapid gradient‐echo (MPRAGE) sequence (repetition time [TR]/inversion time [TI]/echo time [TE] = 2300/900/3.39 ms, flip angle = 9°, field of view = 256 × 256 mm^2^, 160 slices, voxel size = 1.33 × 1.33 × 1 mm^3^) was acquired and used to position the MRS voxel for in vivo scans. Prior to MRS acquisition, *B*_0_ shimming was performed. On both GE MR scanners, *B*_0_ shimming was performed by acquiring field maps from three orthogonal slices centered at the middle of the voxel using the vendor‐supplied shim. On the Siemens standalone MR scanner, shimming was performed using the vendor‐supplied “brain shim”, which relies on a gradient recalled echo‐based sequence for *B*_0_ field mapping and employs second‐order spherical harmonic shims to correct field inhomogeneities. Single voxel MRS was acquired in vivo using the semi‐localized by adiabatic selective refocusing (semi‐LASER) sequence positioned in the posterior cingulate cortex (TR/TE = 2000/35 ms, flip angle = 90°, *N* = 128 transients, spectral bandwidth = 5000 Hz, complex data points = 2048, nominal voxel size = 2 × 2 × 2 cm^3^, variable power and optimized relaxations delays (VAPOR) water suppression with a VAPOR flip angle = 80°) [38]. A non‐water suppressed scan was also acquired with the same parameters but with 4 transients. On both GE scanners, the semi‐LASER implementation is as previously reported [39, 40] and employs 3D outer volume suppression (OVS). On the Siemens MR scanner, the semi‐LASER implementation used sequential refocusing and 1D OVS [41, 42].

**FIGURE 1:**
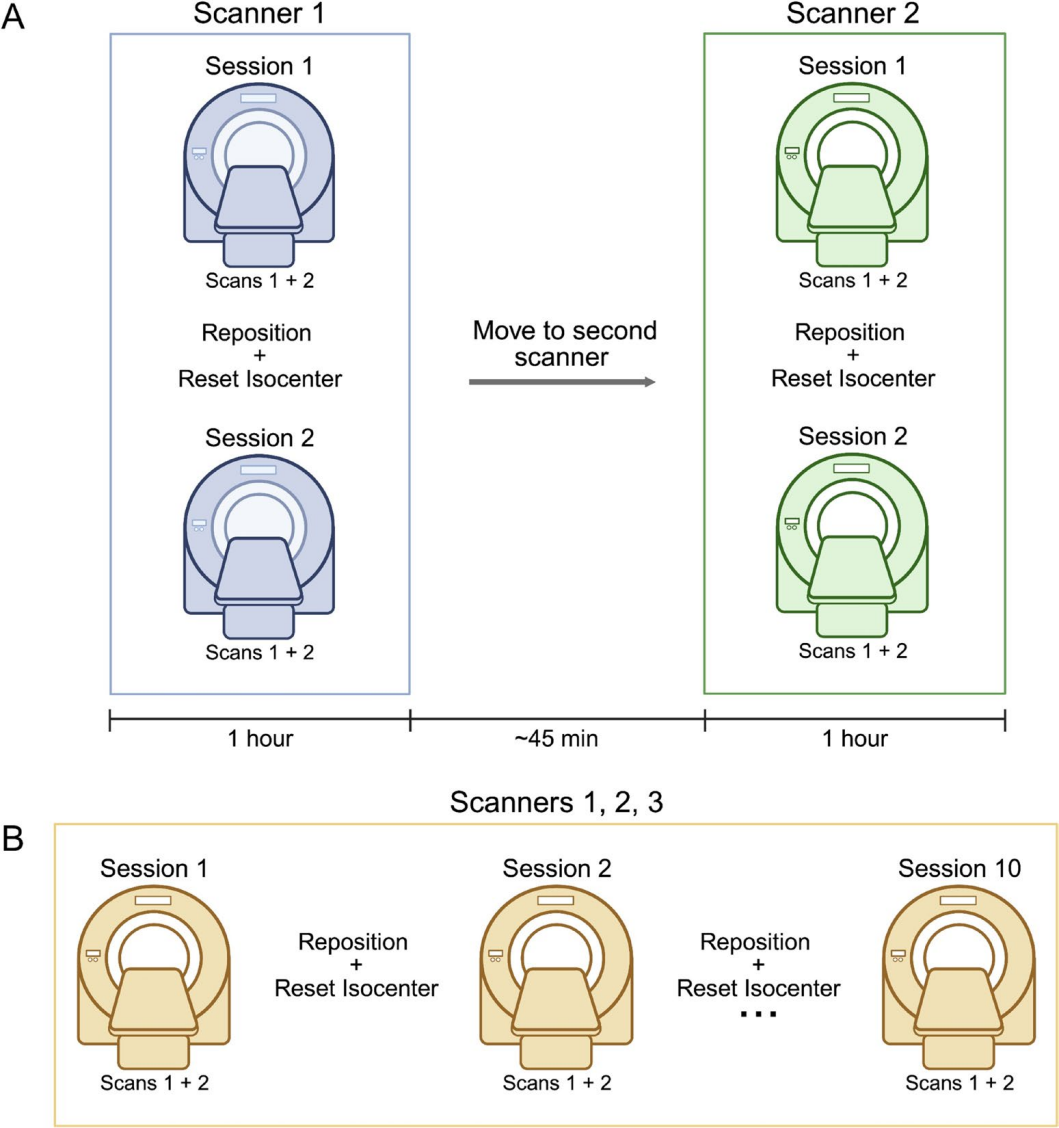
Schematic of repeated MR spectroscopy (MRS) acquisition. (A) In vivo MRS was acquired on the same day (within ~3 hours) from each participant using both the hybrid positron emission tomography (PET)/MR and Siemens standalone MR scanners. Acquisition included two consecutive MRS scans (Session 1) on the first MR scanner. The participant was then removed from the scanner, a new isocenter was set, and two more MRS scans with the same parameters were acquired (Session 2). The participant then moved to the second scanner and the entire acquisition protocol was repeated. The scanner order was randomized (see Table 1). (B) A similar workflow was used for in vitro MRS acquisition but with 10 repeated pairs of scans (10 sessions). In vitro measurements were acquired in the Braino phantom on the hybrid PET/MR and both the Siemens and GE standalone MR scanners.

In vivo scans were acquired from each participant on the same day with the PET/MR and Siemens standalone MR scanners within a span of ~3 hours. Participants were positioned in the scanner for two consecutive MRS scans (i.e., session 1), removed and repositioned, and a new isocenter was set. Two more MRS scans were then acquired (session 2). The subject then moved to the next scanner where the acquisition protocol was repeated (Figure 1). The order in which each participant was scanned first was randomized (Table 1). Robust voxel positioning between session and scanner was facilitated using the voxel overlay on the anatomical images from the first session and the first scanner, and subsequent voxel positioning was matched visually to this position. The PCC voxel location was chosen to facilitate repeated voxel positioning as it is a region easily identified on an anatomical T_1_‐weighted image. Two pairs of spectra (four total scans, two sessions) were acquired for 23 participants at the PET/MR and for 12 participants using the standalone MR scanner. The remaining 11 participants underwent one pair (two consecutive scans, one session) on the Siemens standalone MR scanner. Of note, as the goal was to compare scanner effects and limit biological changes in metabolites which may confound comparisons, both in vivo scans were acquired back‐to‐back on the same day for each participant as these scanners are physically close (across the street). Due to the distance between the PET/MR and the GE standalone MR (~1 hour drive), we were unable to compare in vivo spectra acquired on both GE scanners in the same manner.

**TABLE 1:** Demographics and acquisition details for all participants.

ID	Age (years)	Sex	First acquisition	Standalone MR sessions	PET/MR upgrade
1	21	F	PET/MR	1	Pre
2	27	F	PET/MR	1	Post
3	22	M	PET/MR	2	Post
4	30	F	PET/MR	1	Post
5	22	M	Standalone MR	1	Post
6	22	F	Standalone MR	2	Pre
7	20	F	Standalone MR	2	Post
8	23	F	PET/MR	1	Post
9	21	F	PET/MR	2	Pre
10	22	M	Standalone MR	1	Post
11	30	M	Standalone MR	1	Pre
12	63	F	Standalone MR	2	Post
13	20	M	Standalone MR	2	Pre
14	28	F	Standalone MR	1	Pre
15	21	M	Standalone MR	2	Post
16	19	M	PET/MR	2	Pre
17	20	M	PET/MR	2	Pre
18	34	M	PET/MR	1	Post
19	32	M	Standalone MR	1	Post
20	23	M	PET/MR	2	Post
21	35	F	PET/MR	1	Post
22	20	F	PET/MR	2	Post
23	53	F	standalone MR	2	Post

Abbreviations: F = female; M = male; Post‐upgrade software version = 30.1; Pre‐upgrade software version = 26.003; Standalone MR = Siemens standalone MR scanner.

In vitro MR spectra were acquired using all three scanners (PET/MR, Siemens standalone MR, and GE standalone MR) from the isocenter of the GE Braino phantom (12.5 mM of *N*‐acetylaspartate [NAA], 10 mM of creatine [Cr], 3 mM of choline [Cho], 7.5 mM of myo‐inositol [Ins], 12.5 mM of glutamate [Glu], and 5 mM of lactate [Lac]) [43]. The phantom was positioned in the coil so the isocenter was near or at the center of the phantom in all three directions. Acquisition parameters for in vitro data matched in vivo settings, except *N* = 64 transients and 10 sessions, resulting in 20 spectra per scanner. The GE PET/MR underwent a software upgrade midway through data collection. To evaluate the effect of this upgrade, 10 pairs of in vitro spectra were collected using the same protocol before and after the upgrade.

### 2.3 MR Data Analysis

Spectra were fitted using LCModel (v6.3‐*1R*) from 4.0 to −1 ppm with water scaling and eddy current correction [44]. The spectra were fit with an extended upfield region to generate a noise‐only spectral region for SNR calculations. A GAMMA‐simulated basis set consisting of 27 metabolites (alanine, aspartate, methyl Cr, methylene Cr, phosphocreatine (PCr), gamma‐aminobutyric acid, glucose, glutamine, Glu, glycerophosphocholine (GPC), phosphocholine (PCh), glutathione, Ins, Lac, NAA, *N*‐acetyl‐aspartyl‐glutamate (NAAG), scyllo‐inositol, taurine, four lipid resonances, and five macromolecular resonances) was used for spectral fitting. Metabolites with Cramer‐Rao lower bounds (CRLBs) > 20% were evaluated further.

Spectral quality metrics were determined using MATLAB (vR2023a, MathWorks). Spectral quality was assessed using SNR and full width at half maximum (FWHM) in Hz of the fitted NAA peak. SNR was defined as the maximum signal of the fitted NAA peak from 1.9 to 2.1 ppm divided by the SD of a noise‐only region of the phased spectrum (PET/MR: −0.11 to −1.0 ppm, Siemens standalone MR scanner: −0.07 to −0.99 ppm, GE standalone MR scanner: −0.11 to −1.0 ppm). Noise regions used the last 93 data points from all spectra, regardless of the scanner. FWHM was calculated as the width at half the height of the fitted NAA peak. Metabolite concentrations evaluated in vivo include total *N‐*acetylaspartate + *N‐*acetyl‐aspartyl‐glutamate (tNAA), total GPC + PCh (tCho), Glu, and Ins, all normalized to total Cr + PCr (tCr). In vitro, metabolite concentrations including NAA, Cho, Glu, Ins, and Lac, normalized to Cr, were evaluated. In vitro spectra were excluded if FWHM was > 10 Hz for the NAA peak. Complete details for MRS acquisition and analysis are reported in the MRSinMRS checklist (Supporting Information: Appendix 1) [45].

### 2.4 Statistical Analysis

Statistical analysis was performed in R. In vivo data were compared between two MR scanners (PET/MR and Siemens standalone MR) using mixed models in which the outcome was FWHM, SNR, or metabolite concentration with scanner as a fixed effect and three random effects (subject, scanner nested in subject, and session nested in scanner and subject). In vitro data were compared across three MR scanners (PET/MR, Siemens standalone MR, and GE standalone MR) using the same approach. To determine the impact of the software upgrade of the PET/MR on SNR, FWHM, and metabolite concentrations, we subset to the PET/MR observations and fit mixed models with an indicator variable for software upgrade as a fixed effect and two random effects (subject and subject‐session). PET/MR intraclass correlation coefficients (ICCs) were calculated using the same mixed model (described below). To examine repeatability in the Siemens standalone MR scanner, we subset to the Siemens standalone MR data and fit mixed models with subject and subject‐session random effects. Repeatability was calculated using three variance components: the subject‐session random effect variance, $\sigma_{\mathrm{sp}}^{2}$; the subject random effect variance, $\sigma_{s}^{2}$; and the residual variance, $\sigma_{e}^{2}$. Let $b_{i}$ and $c_{\mathrm{ij}}$ denote the random effects of subject *i* and session *j*. Repeatability within‐session was calculated as.

(1) $$R_{w}=\frac{\sigma_{\mathrm{sp}}^{2}+\sigma_{s}^{2}+2\mathrm{cov}\left(b_{i},c_{\mathrm{ij}}\right)}{\sigma_{\mathrm{sp}}^{2}+\sigma_{s}^{2}+2\mathrm{cov}\left(b_{i},c_{\mathrm{ij}}\right)+\sigma_{e}^{2}}$$

While between‐session repeatability was calculated as.

(2) $$R_{b}=\frac{\sigma_{s}^{2}+2\mathrm{cov}\left(b_{i},c_{\mathrm{ij}}\right)}{\sigma_{\mathrm{sp}}^{2}+\sigma_{s}^{2}+2\mathrm{cov}\left(b_{i},c_{\mathrm{ij}}\right)+\sigma_{e}^{2}}$$

ICC values were categorized as excellent (> 0.90), good (0.75–0.90), moderate (0.5–0.74), and poor (< 0.5) [46]. Statistical significance (i.e., *p* values) was not determined for ICC values, but the categories above indicate the strength of correlation between repeated measurements.

For in vitro data, mean squared error (MSE) was calculated as the mean of the squared difference between the estimated and true concentrations, pooled across the 10 scan pairs per scanner. MSE was decomposed into squared bias (squared difference between the average measured concentration and the true concentration) and variance (mean squared difference between each measurement and the mean of the measurements) to disentangle the sources of errors (MSE = bias^2^ + variance). All spectral quality metrics and metabolite concentrations are reported within the text as the mean ± standard deviation (SD) across all sessions and scans unless otherwise stated. Of note, model estimates and standard error from the mixed models are reported in the tables, and these estimates may differ slightly from the mean ± SD values reported within the text. Significance was determined by *p* ≤ 0.05 for all analyses.

## 3 Results

**FIGURE 2:**
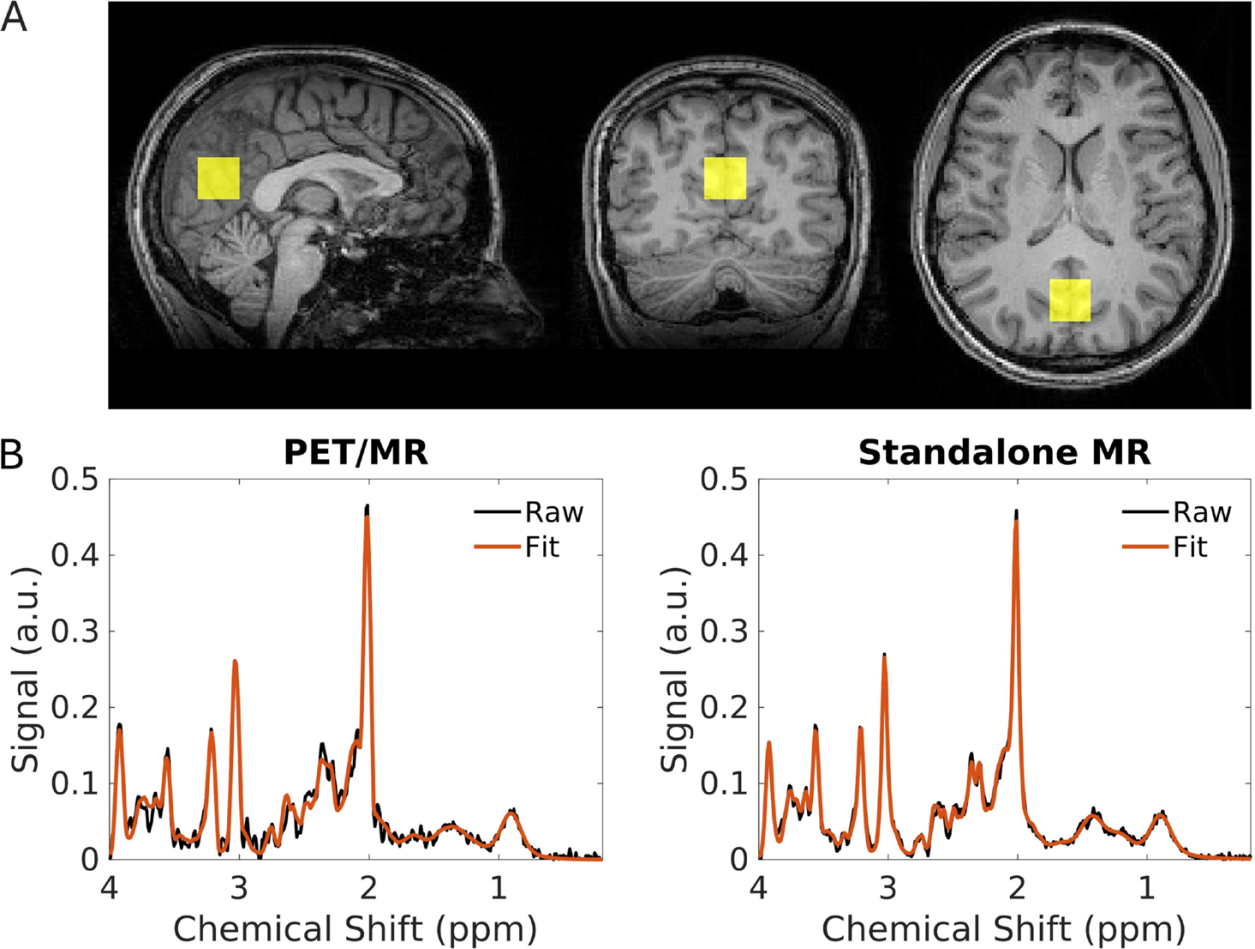
Representative voxel positioning and corresponding MR spectra. (A) Single voxel positioning (yellow square) in the posterior cingulate cortex overlaid on a *T*_1_‐weighted image in the sagittal, coronal, and axial planes. Images are displayed in radiological orientation. (B) Corresponding in vivo MR spectra acquired using the hybrid GE positron emission tomography (PET)/MR and Siemens standalone MR scanners in a healthy volunteer (female, 20 years old).

**FIGURE 3:**
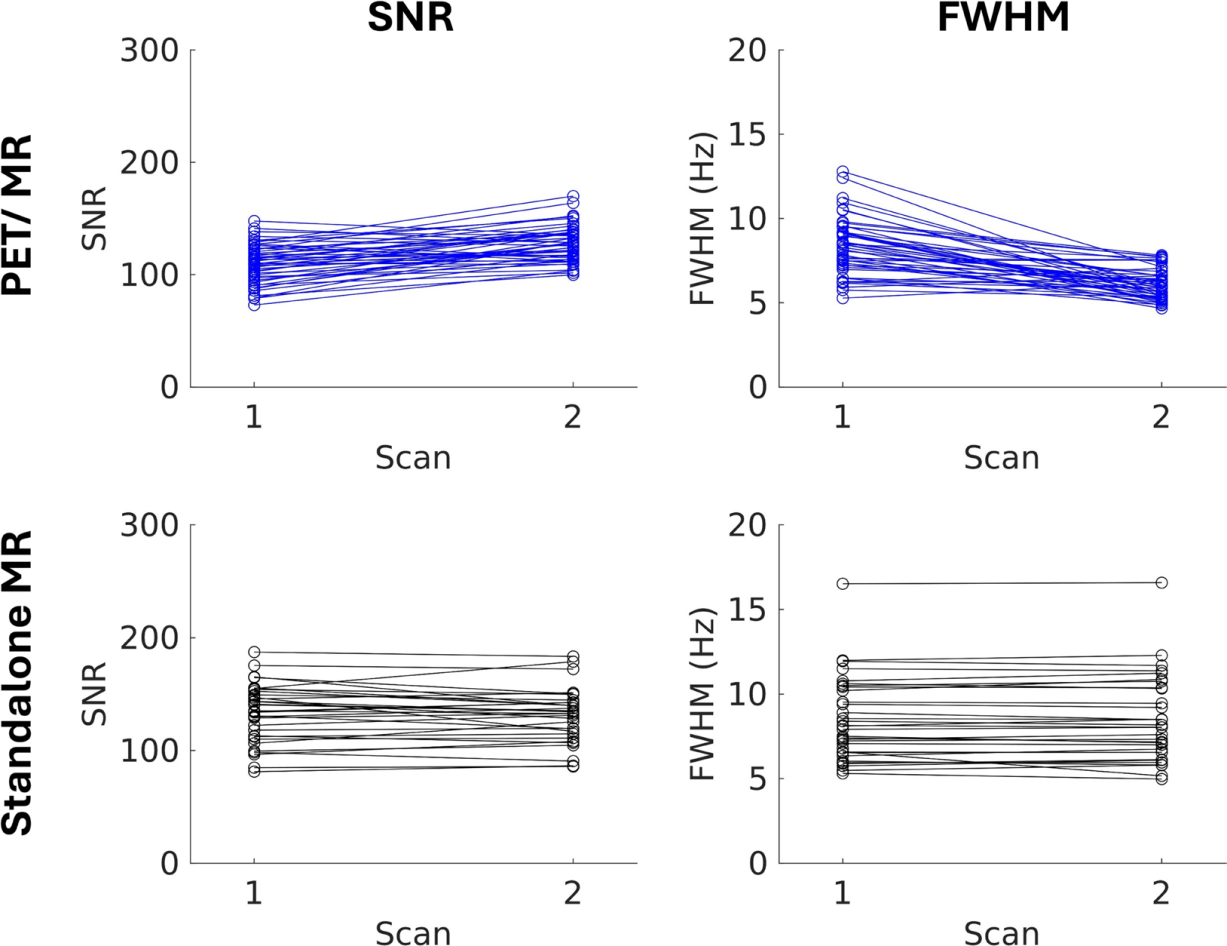
Comparison of signal‐to‐noise ratio (SNR) and full width at half maximum (FWHM) from repeated spectra acquired in healthy volunteers. Metrics are shown for MR spectra acquired using a hybrid GE positron emission tomography (PET)/MR (top) and a Siemens standalone MR scanner (bottom). Participants underwent two sessions, with two consecutive scans (scan 1 and 2) acquired within each session. Lines represent individual sessions acquired for each participant.

Of 24 healthy participants enrolled, 23 were included in the final cohort (mean ± SD age = 27 ± 11 years; 11 male and 12 female; Table 1). One participant was excluded due to claustrophobia, which prevented them from completing the MR scans. Data acquired using the Siemens standalone MR scanner for a separate participant were excluded due to excessive motion during the MR acquisition, resulting in unusable spectral data. Representative voxel positioning and in vivo MR spectra acquired using both the hybrid PET/MR and the Siemens standalone MR scanners are shown in Figure 2. In vivo SNR and FWHM for spectra acquired on both scanners are presented in Figure 3 and Table 2. For spectra acquired in healthy volunteers, SNR was significantly lower on PET/MR (118.8 ± 19.1) compared to the Siemens standalone MR scanner (132.2 ± 24.4) (Table 2). Conversely, FWHM was significantly narrower on the PET/MR (7.2 ± 1.8 Hz) compared to the Siemens standalone MR scanner (8.4 ± 2.5 Hz). Metabolite concentrations normalized to tCr are reported in Table 2. No metabolites were excluded due to CRLBs > 20%. All metabolite concentrations normalized to tCr were significantly higher in spectra acquired with the Siemens standalone MR scanner compared to the PET/MR (Table 2).

**TABLE 2:** Hierarchical linear mixed model comparing in vivo metrics and metabolite concentrations from spectra acquired on hybrid PET/MR and Siemens standalone MR scanners.

Metric	Scanner	Estimate^a^	Standard error	Degrees of freedom	*t*‐statistic	*p*
SNR	PET/MR	118.8	3.4	35.0	3.1	0.0052
	Standalone MR	131.8	4.2	22.7		
FWHM	PET/MR	7.2	0.3	37.8	2.5	0.018
	Standalone MR	8.4	0.5	42.7		
tNAA/tCr	PET/MR	1.60	0.03	24.9	4.3	0.00028
	Standalone MR	1.66	0.01	21.6		
tCho/tCr	PET/MR	0.21	< 0.01	24.5	14.0	< 0.0001
	Standalone MR	0.24	< 0.01	20.1		
Glu/tCr	PET/MR	1.25	0.02	29.7	3.3	0.0037
	Standalone MR	1.31	0.02	21.3		
Ins/tCr	PET/MR	0.69	0.01	29.4	24.0	< 0.0001
	Standalone MR	0.91	< 0.01	21.0		

*Note*: Standard errors and degrees of freedom were obtained using the Satterthwaite approximation.

Abbreviations: FWHM = full width at half maximum; Glu = glutamate; Ins = myo‐inositol; SNR = signal‐to‐noise ratio; Standalone MR = Siemens standalone MR scanner; tCho = total glycerophosphocholine + phosphocholine; tCr = total creatine + phosphocreatine; tNAA = total *N*‐acetylaspartate + *N*‐acetyl‐aspartyl‐glutamate.

^a^ Estimates reflect mixed model estimates, with PET/MR values used as the reference.

ICC values indicated moderate to excellent repeatability for all metabolites quantified from in vivo spectra acquired on both scanners (Table 3). Within‐session repeatability was higher for tNAA/tCr, tCho/tCr, and Glu/tCr quantified from spectra acquired using the Siemens standalone MR scanner, whereas between‐session repeatability was higher for tNAA/tCr, Glu/tCr, and Ins/tCr using the PET/MR. The software upgrade on the PET/MR did not significantly impact in vivo SNR (pre‐upgrade: 116.6 ± 18.6, post‐upgrade: 120.0 ± 19.4, *t* = 0.7, *p* = 0.51), FWHM (pre‐upgrade: 7.0 ± 1.9 Hz, post‐upgrade: 7.3 ± 1.7 Hz, *t* = 1.0, *p* = 0.31), or quantification of tNAA/tCr (pre‐upgrade: 1.58 ± 0.10, post‐upgrade: 1.61 ± 0.15, *t* = 0.5, *p* = 0.59) and Ins/tCr (pre‐upgrade: 0.67 ± 0.03, post‐upgrade: 0.70 ± 0.06, *t* = 1.5, *p* = 0.14) (Table S1). Compared to spectra acquired pre‐upgrade on the PET/MR, spectra acquired post‐upgrade had significantly higher Glu/tCr (pre‐upgrade: 1.18 ± 0.07, post‐upgrade: 1.28 ± 0.10) and tCho/tCr (pre‐upgrade: 0.20 ± < 0.01, post‐upgrade: 0.22 ± 0.02); however, these comparisons are across different groups of participants (Table S1).

**TABLE 3:** Intraclass correlation coefficients indicating within‐session (*R*_w_) and between‐session (*R*_b_) repeatability of metabolite quantification in healthy volunteers.

Metabolite	PET/MR		Standalone MR	
	*R*_w_	*R*_b_	*R*_w_	*R*_b_
tNAA/tCr	0.914	0.914	0.950	0.725
tCho/tCr	0.791	0.724	0.917	0.760
Glu/tCr	0.838	0.804	0.911	0.793
Ins/tCr	0.890	0.848	0.882	0.700

Abbreviations: Glu = glutamate; Ins = myo‐inositol; Standalone MR = Siemens standalone MR scanner; tCho = total glycerophosphocholine + phosphocholine; tCr = total creatine + phosphocreatine; tNAA = total *N*‐acetylaspartate + *N*‐acetyl‐aspartyl‐glutamate.

**FIGURE 4:**
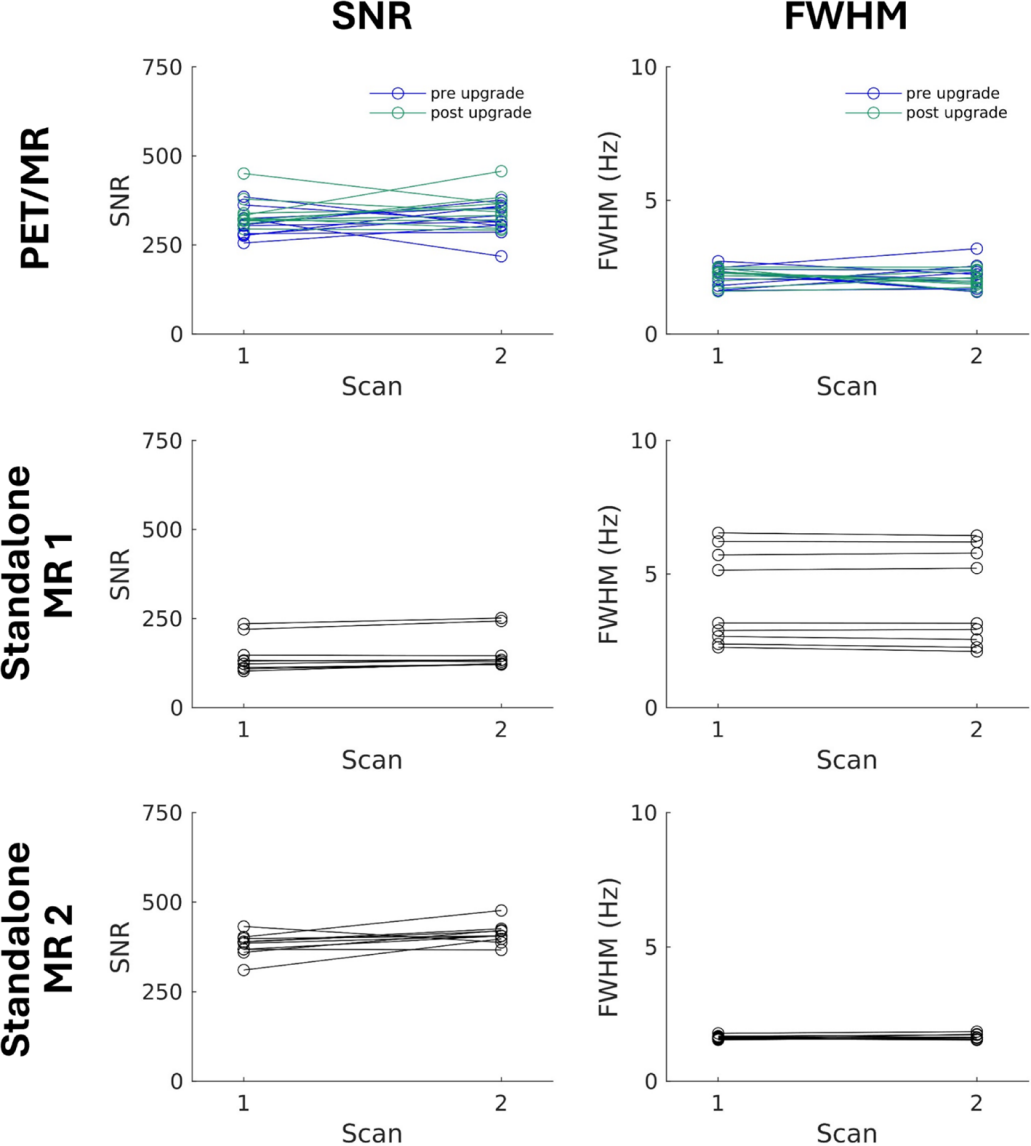
Comparison of signal‐to‐noise ratio (SNR) and full width at half maximum (FWHM) from repeated in vitro (phantom) spectra. MR spectra were acquired using a hybrid positron emission tomography (PET)/MR (top), a Siemens standalone MR scanner (Standalone MR 1, middle) and GE standalone MR scanner (Standalone MR 2, bottom). Within each session, a pair of scans (scan 1 and 2) were acquired consecutively. Ten sessions were acquired on each scanner, including 10 pre‐upgrade (blue) and 10 post‐upgrade (green) on the hybrid PET/MR scanner. Lines represent individual sessions.

For in vitro scans acquired in the Braino phantom, SNR and FWHM values are displayed in Figure 4. One pair of spectra acquired on the Siemens standalone MR scanner was excluded due to FWHM. SNR of in vitro spectra acquired using the PET/MR (329.1 ± 46.1) was significantly higher than the Siemens standalone MR scanner (150.8 ± 49.3) but significantly lower than the GE standalone MR scanner (395.9 ± 33.7) (Table 4). Similarly, FWHM from spectra acquired on the PET/MR (2.1 ± 0.4 Hz) was significantly narrower than spectra acquired with the Siemens standalone MR scanner (4.1 ± 1.7 Hz). Compared to the GE standalone MR scanner (1.6 ± 0.1 Hz), FWHM was not significantly different than spectra acquired on the PET/MR (*t* = −1.5, *p* = 0.14). Overall, spectra acquired on the PET/MR had lower MSE than the Siemens standalone MR scanner for NAA/Cr (PET/MR: 0.00160, Siemens standalone MR scanner: 0.00238), Cho/Cr (PET/MR: 0.00009, Siemens standalone MR scanner: 0.00103), and Lac/Cr (PET/MR: 0.01752, Siemens standalone MR scanner: 0.03446), but lower accuracy for Glu/Cr (PET/MR: 0.12070, Siemens standalone MR scanner: 0.02486) and Ins/Cr (PET/MR: 0.02909, Siemens standalone MR scanner: 0.00183). Compared to the PET/MR, the GE standalone MR scanner had higher MSE for NAA/Cr (0.00176), Ins/Cr (0.03383), and Glu/Cr (0.14253), yet lower MSE for Cho/Cr (0.00005) and Lac/Cr (0.01493) (Table 5).

**TABLE 4:** Hierarchical linear mixed model comparing in vitro metrics and metabolite concentrations from spectra acquired on hybrid PET/MR, Siemens standalone, and GE standalone MR scanners.

Metric	Scanner	Estimate^a^	Standard error	Degrees of freedom	*t*‐statistic	*p*
SNR	PET/MR	329.1	8.2	36.0	—	—
	Standalone MR 1	150.8	14.8	36.0	−12.1	< 0.0001
	Standalone MR 2	395.9	14.3	36.0	4.7	< 0.0001
FWHM (Hz)	PET/MR	2.1	0.2	36.0	—	—
	Standalone MR 1	4.1	0.3	36.0	5.6	< 0.0001
	Standalone MR 2	1.6	0.3	36.0	−1.5	0.14
NAA/Cr	PET/MR	1.22	< 0.01	36.0	—	—
	Standalone MR 1	1.21	< 0.01	36.0	−1.0	0.33
	Standalone MR 2	1.21	< 0.01	36.0	−0.2	0.81
Cho/Cr	PET/MR	0.31	< 0.01	36.0	—	—
	Standalone MR 1	0.33	< 0.01	36.0	12.6	< 0.0001
	Standalone MR 2	0.31	< 0.01	36.0	−1.6	0.12
Glu/Cr	PET/MR	0.90	< 0.01	36.0	—	—
	Standalone MR 1	1.10	0.01	36.0	15.6	< 0.0001
	Standalone MR 2	0.87	0.01	36.0	−2.7	0.011
Ins/Cr	PET/MR	0.58	< 0.01	36.0	—	—
	Standalone MR 1	0.78	< 0.01	36.0	25.5	< 0.0001
	Standalone MR 2	0.57	< 0.01	36.0	−1.9	0.072
Lac/Cr	PET/MR	0.37	< 0.01	36.0	—	—
	Standalone MR 1	0.32	< 0.01	36.0	−5.1	< 0.0001
	Standalone MR 2	0.38	< 0.01	36.0	1.1	0.28

*Note*: Standard errors and degrees of freedom were obtained using the Satterthwaite approximation.

Abbreviations: Cho = choline; Cr = creatine; FWHM = full width at half maximum; Glu = glutamate; Ins = myo‐inositol; Lac = lactate; NAA = *N*‐acetylaspartate; SNR = signal‐to‐noise ratio; Standalone MR 1 = Siemens standalone MR scanner; Standalone MR 2 = GE standalone MR scanner.

^a^ Estimates reflect mixed model estimates, with PET/MR values used as the reference.

**TABLE 5:** Mean squared error (MSE) values, decomposed into bias^2^ and variance, for metabolite quantification from spectra acquired in vitro.

Metabolite	PET/MR			Standalone MR 1			Standalone MR 2		
	Bias^2^	Variance	MSE	Bias^2^	Variance	MSE	Bias^2^	Variance	MSE
NAA/Cr	0.00119	0.00041	0.00160	0.00174	0.00065	0.00238	0.00131	0.00045	0.00176
Cho/Cr	0.00008	0.00001	0.00009	0.00097	0.00006	0.00103	0.00004	0.00001	0.00005
Glu/Cr	0.11942	0.00128	0.12070	0.02372	0.00114	0.02486	0.14232	0.00021	0.14253
Ins/Cr	0.02887	0.00021	0.02909	0.00074	0.00109	0.00183	0.03378	0.00004	0.03383
Lac/Cr	0.01741	0.00011	0.01752	0.03242	0.00204	0.03446	0.01490	0.00003	0.01493

Abbreviations: Cho = choline; Cr = creatine; Glu = glutamate; Ins = myo‐inositol; Lac = lactate; MSE = mean squared error; NAA = *N*‐acetylaspartate; Standalone MR 1 = Siemens standalone MR scanner; Standalone MR 2 = GE standalone MR scanner.

The PET/MR software upgrade did not significantly affect in vitro FWHM (pre‐upgrade: 2.2 ± 0.4 Hz, post‐upgrade: 2.1 ± 0.3 Hz, *t* = −0.4, *p* = 0.70); however, SNR increased significantly in spectra acquired post‐upgrade (344.9 ± 45.7) compared to pre‐upgrade (313.2 ± 41.7). Metabolite quantification did not differ significantly pre‐upgrade to post‐upgrade for NAA/Cr (pre‐upgrade: 1.21 ± 0.02, post‐upgrade: 1.22 ± 0.02, *t* = 0.7, *p* = 0.46), Cho/Cr (pre‐upgrade: 0.31 ± < 0.01, post‐upgrade: 0.31 ± < 0.01, *t* = 0.1, *p* = 0.95), or Lac/Cr (pre‐upgrade: 0.37 ± 0.01, post‐upgrade: 0.37 ± < 0.01, *t* = −0.5, *p* = 0.66), but significant increases were observed for Ins/Cr (pre‐upgrade: 0.57 ± < 0.01, post‐upgrade: 0.59 ± < 0.01) and Glu/Cr (pre‐upgrade: 0.87 ± 0.02, post‐upgrade: 0.94 ± 0.02) (Table S2). Decreases in MSE for Ins/Cr and Glu/Cr were observed following the software upgrade, but regardless of software version, MSE for both Ins/Cr and Glu/Cr in the PET/MR were higher than MSE quantified using the Siemens standalone MR scanner and similar to the GE standalone MR scanner (Ins/Cr, pre‐upgrade: 0.03351, post‐upgrade: 0.02467, Siemens standalone MR scanner: 0.00183, GE standalone MR scanner: 0.03383; Glu/Cr, pre‐upgrade: 0.14251, post‐upgrade: 0.09889, Siemens standalone MR scanner: 0.02486, GE standalone MR scanner: 0.14253).

## 4 Discussion

Results from this study may suggest spectra acquired in healthy volunteers and the Braino phantom in the presence of the PET detector do not hinder MR spectral quality for data acquired using a hybrid PET/MR scanner. While the Siemens standalone MR scanner generated in vivo spectra with higher SNR compared to the PET/MR, spectra acquired with the PET/MR had narrower spectral bandwidth, similar within‐session repeatability for metabolite estimations, and higher between‐session repeatability. In vitro spectra acquired with the PET/MR had significantly higher SNR than the Siemens standalone MR scanner but lower SNR than the GE standalone MR scanner. The PET/MR also resulted in higher accuracy of metabolite quantification for some metabolites. Differences in quality for spectra acquired in vivo versus in vitro are attributed primarily to differences in coils and voxel position, as the RF receive coils used on both GE scanners have fewer posterior coil elements compared to the RF coil used on the Siemens standalone MR scanner. This is one explanation for the higher in vivo SNR of spectra acquired using the Siemens standalone MR scanner (acquired in a posterior region of the brain), a trend not observed for in vitro spectra (acquired in the isocenter). Broader in vivo and in vitro FWHM for spectra acquired using the Siemens standalone MR scanner is potentially due to hardware, as the magnet in this scanner is older than both of the GE scanners (PET/MR and standalone MR). There is also a difference in the semi‐LASER implementation on the GE and Siemens scanners (see Methods), which may be responsible for some of the differences in both spectral metrics as well as the higher Ins/tCr concentration observed for all spectra acquired on the Siemens standalone MR scanner. Notwithstanding, high overall spectral quality and reproducibility of metabolite quantification support robust MR spectra are feasible when using a hybrid PET/MR for MRS acquisition.

Prior studies evaluated the impact of PET detectors, either as a PET insert or within a 3T or 7T hybrid PET/MR scanner, on spin echo, gradient echo, and echo planar imaging sequences [21, 22, 23]. All studies reported the presence of the PET detector had minimal effect on MR imaging metrics including SNR, field homogeneity, and image quality. MR performance was reported to be largely equivalent to that of a standalone scanner of the same field strength. Additional work using a preclinical 3T MR scanner with a 24 cm bore assessed MR data quality with the PET insert positioned outside the bore, inside the bore, and during simultaneous PET/MR acquisition. *B*_0_ field homogeneity, *T*_1_, and *T*_2_ relaxation times were unaffected by the presence of the PET insert across all conditions [47]. These prior findings suggest MR imaging metrics are not necessarily impacted by the PET detector in hybrid PET/MR scanners [21, 22, 23, 47].

Quantification of metabolite concentrations from spectra acquired with a hybrid PET/MR scanner is a largely understudied parameter in prior work. The results from the present study suggest in vitro acquisition with the PET/MR results in comparable quantification of metabolite concentrations. In vivo, however, all metabolite concentrations were higher for spectra acquired on the standalone MR scanner, highlighting the importance of standardization when using different scanners or using the same scanner for all participants within a single study. For repeated measurements in vivo, the standalone MR scanner demonstrated better repeatability compared to the hybrid PET/MR under the same conditions and parameters (i.e., within‐session measurements). For in vivo measurements, between sessions repeatability was generally better for spectra acquired with the PET/MR for most metabolites, suggesting more consistent quantification when conditions vary across data acquisition from the same participant. Importantly, both scanners demonstrated good to excellent repeatability within and between sessions for all metabolites quantified in vivo, along with relatively low MSE, indicating both scanners consistently facilitate repeatable and robust metabolite quantification. For studies requiring multiple scans across different days, these results suggest the use of a hybrid PET/MR for metabolite quantification is comparable to a standalone MR scanner of the same field strength.

Evaluation of spectral metrics in vitro revealed comparable spectral quality across scanners, though the Siemens standalone MR scanner resulted in spectra with decreased SNR and increased FWHM compared to the PET/MR. MSE provided a method of absolute comparison of metabolites in vitro using the known concentration of the phantom as opposed to the relative comparisons between scanners used for in vivo analysis. In addition to the decreased SNR and FWHM, the Siemens standalone MR scanner also exhibited increased MSE for some metabolites when compared to the PET/MR. Prior comparisons of Siemens and GE standalone MR scanners revealed differences in FWHM and metabolite quantification compared to the present study [39], suggesting differences in sites or scanners (i.e., age of the magnet) may be responsible for some of the differences observed.

### 4.1 Limitations

The primary limitation of this comparison study is the two MR scanners used for in vivo data acquisition are from different vendors and rely on different RF receive coils, introducing intrinsic hardware and software differences which may independently impact data acquisition and the quality of spectra. This was partially addressed by including a standalone MR scanner from the same vendor as the hybrid PET/MR for in vitro data acquisition. Notably, the PET/MR scanner is not equipped with higher‐order shims that are present on both standalone MR scanners; however, this did not appear to degrade spectral quality or result in broader linewidths. Evaluation was limited to a single cohort at a single site using one field strength (3T). The semi‐LASER sequence used on the GE compared to Siemens MR scanners differs slightly in both the pulses and OVS, which could contribute to the differences observed in data and in metabolite quantification. For all scans, only a single voxel region was evaluated, and future investigation using deep brain voxels, oblique voxels, or multi‐voxel techniques is warranted. The small sample size, primarily composed of young healthy adults without pathology, may further limit the generalizability of the results. Future studies would benefit from evaluation of patient cohorts, additional scanner vendors (both standalone and hybrid MR scanners), and other MR field strengths to extend these findings.

## 5 Conclusion

MR spectral quality is not diminished when using a hybrid PET/MR scanner. Comparable metrics of spectral quality, accuracy, and repeatability of metabolite quantification were reported for spectra acquired with both hybrid and standalone MR scanners. As hybrid scanners expand into larger clinical markets, these findings indicate robust MRS data may be acquired using hybrid PET/MR scanners.

## Disclosure

Drs. Sagar Mandava and Ralph Noeske are employees of GE HealthCare. Figure 1 was created in BioRender: Zuleger, T. (2025), https://BioRender.com/g406011.

## Supporting information


**Table S1:** Hierarchical linear mixed model comparing in vivo metrics and metabolite concentrations from spectra acquired on hybrid PET/MR pre‐upgrade to post‐upgrade.
**Table S2:** Hierarchical linear mixed model comparing in vitro metrics and metabolite concentrations from spectra acquired on hybrid PET/MR pre‐upgrade to post‐upgrade.

## References

[1] S. Musafargani , K. K. Ghosh , S. Mishra , P. Mahalakshmi , P. Padmanabhan , and B. Gulyás , “PET/MRI: A Frontier in Era of Complementary Hybrid Imaging,” European Journal of Hybrid Imaging 2, no. 1 (2018): 12. 29998214 10.1186/s41824-018-0030-6 PMC6015803

[2] Z. Chen , S. D. Jamadar , S. Li , et al., “From Simultaneous to Synergistic MR‐PET Brain Imaging: A Review of Hybrid MR‐PET Imaging Methodologies,” Human Brain Mapping 39, no. 12 (2018): 5126–5144. 30076750 10.1002/hbm.24314 PMC6866605

[3] B. Sattler , “Clinical Molecular PET/MRI Hybrid Imaging,” in Handbook of Nuclear Medicine and Molecular Imaging for Physicists: Instrumentation and Imaging Procedures, vol. 1, ed. M. Ljungberg (CRC Press, 2022), 397–426.

[4] L. Evangelista , P. Artoli , P. Bartoletti , et al., “Clinical Application of PET/MRI,” in Radiology‐Nuclear Medicine Diagnostic Imaging, 1st ed., ed. A. Gholamrezanezhad , M. Assadi , and H. Jadvar (Wiley Blackwell, 2023), 788–813.

[5] J. G. Mannheim , A. M. Schmid , J. Schwenck , et al., “PET/MRI Hybrid Systems,” Seminars in Nuclear Medicine 48, no. 4 (2018): 332–347. 29852943 10.1053/j.semnuclmed.2018.02.011

[6] P. Sabeghi , S. Katal , M. Chen , et al., “Update on Positron Emission Tomography/Magnetic Resonance Imaging: Cancer and Inflammation Imaging in the Clinic,” Magnetic Resonance Imaging Clinics of North America 31, no. 4 (2023): 517–538. 37741639 10.1016/j.mric.2023.07.001

[7] F. S. Furtado , M. Hesami , S. McDermott , H. Kulkarni , A. Herold , and O. A. Catalano , “The Synergistic Effect of PET/MRI in Whole‐Body Oncologic Imaging: An Expert Review,” Clinical and Translational Imaging 11, no. 4 (2023): 351–364.

[8] L. Evangelista , F. Zattoni , G. Cassarino , et al., “PET/MRI in Prostate Cancer: A Systematic Review and Meta‐Analysis,” European Journal of Nuclear Medicine and Molecular Imaging 48, no. 3 (2021): 859–873. 32901351 10.1007/s00259-020-05025-0 PMC8036222

[9] Y. Ming , N. Wu , T. Qian , et al., “Progress and Future Trends in PET/CT and PET/MRI Molecular Imaging Approaches for Breast Cancer,” Frontiers in Oncology 10 (2020): 1301, 10.3389/fonc.2020.01301. 32903496 PMC7435066

[10] A. M. Fowler and R. M. Strigel , “Clinical Advances in PET‐MRI for Breast Cancer,” Lancet Oncology 23, no. 1 (2022): e32–e43. 34973230 10.1016/S1470-2045(21)00577-5 PMC9673821

[11] L. Lindenberg , M. Ahlman , B. Turkbey , E. Mena , and P. Choyke , “Advancement of MR and PET/MR in Prostate Cancer,” Seminars in Nuclear Medicine 46, no. 6 (2016): 536–543. 27825433 10.1053/j.semnuclmed.2016.07.001

[12] U. Metser , A. Berlin , J. Halankar , et al., “18F‐Fluorocholine PET Whole‐Body MRI in the Staging of High‐Risk Prostate Cancer,” American Journal of Roentgenology 210, no. 3 (2018): 635–640. 29323548 10.2214/AJR.17.18567

[13] M. Tóth , P. Barsi , Z. Tóth , et al., “The Role of Hybrid FDG‐PET/MRI on Decision‐Making in Presurgical Evaluation of Drug‐Resistant Epilepsy,” BMC Neurology 21, no. 1 (2021): 363. 34537017 10.1186/s12883-021-02352-z PMC8449490

[14] S. Shang , D. Li , Y. Tian , et al., “Hybrid PET‐MRI for Early Detection of Dopaminergic Dysfunction and Microstructural Degradation Involved in Parkinson's Disease,” Communications Biology 4, no. 1 (2021): 1162. 34621005 10.1038/s42003-021-02705-x PMC8497575

[15] J. Dukart , K. Mueller , A. Horstmann , et al., “Combined Evaluation of FDG‐PET and MRI Improves Detection and Differentiation of Dementia,” PLoS One 6, no. 3 (2011): e18111. 21448435 10.1371/journal.pone.0018111 PMC3063183

[16] J. A. Disselhorst , I. Bezrukov , A. Kolb , C. Parl , and B. J. Pichler , “Principles of PET/MR Imaging,” Journal of Nuclear Medicine 55, no. Supplement 2 (2014): 2S–10S. 24819419 10.2967/jnumed.113.129098

[17] M. S. H. Akram , T. Obata , M. Suga , et al., “MRI Compatibility Study of an Integrated PET/RF‐Coil Prototype System at 3T,” Journal of Magnetic Resonance 283 (2017): 62–70. 28881235 10.1016/j.jmr.2017.08.010

[18] S. Vandenberghe and P. K. Marsden , “PET‐MRI: A Review of Challenges and Solutions in the Development of Integrated Multimodality Imaging,” Physics in Medicine and Biology 60, no. 4 (2015): R115. 25650582 10.1088/0031-9155/60/4/R115

[19] G. Delso and S. Ziegler , “PET/MRI System Design,” European Journal of Nuclear Medicine and Molecular Imaging 36, no. 1 (2009): 86–92, 10.1007/s00259-008-1008-6. 19104809

[20] P. Galve , B. Rodriguez‐Vila , J. L. Herraiz , et al., “Recent Advances in Combined Positron Emission Tomography and Magnetic Resonance Imaging,” Journal of Instrumentation 19, no. 1 (2024): C01001.

[21] F. P. Schmidt , M. S. Allen , R. Ladebeck , et al., “Evaluation of the MRI Compatibility of PET Detectors Modules for Organ‐Specific Inserts in a 3T and 7T MRI Scanner,” Medical Physics 51, no. 2 (2024): 991–1006. 38150577 10.1002/mp.16923 PMC10923015

[22] G. Delso , S. Fürst , B. Jakoby , et al., “Performance Measurements of the Siemens mMR Integrated Whole‐Body PET/MR Scanner,” Journal of Nuclear Medicine 52, no. 12 (2011): 1914–1922. 22080447 10.2967/jnumed.111.092726

[23] A. Kolb , H. F. Wehrl , M. Hofmann , et al., “Technical Performance Evaluation of a Human Brain PET/MRI System,” European Radiology 22, no. 8 (2012): 1776–1788. 22752524 10.1007/s00330-012-2415-4

[24] A. Bhattacharya and C. C. Fleischer , Evaluation of Repeated Magnetic Resonance Spectroscopy Data Acquired In Vivo Using a Hybrid PET/MRI Scanner Compared to a Standalone MRI Scanner, vol. 1 (ISMRM MRS Workshop, 2024).

[25] A. Bhattacharya , B. B. Risk , and C. C. Fleischer , “Comparison of Magnetic Resonance Spectroscopy Data Acquired With Hybrid PET‐MRI and Standalone MRI Scanners,” Proceedings of the International Society for Magnetic Resonance in Medicine 31 (2023): 3859.

[26] A. C. Pollard , J. de la Cerda , F. W. Schuler , C. V. Kingsley , S. T. Gammon , and M. D. Pagel , “Evaluations of the Performances of PET and MRI in a Simultaneous PET/MRI Instrument for Pre‐Clinical Imaging,” EJNMMI Physics 9, no. 1 (2022): 70. 36209262 10.1186/s40658-022-00483-x PMC9547760

[27] V. Panebianco , F. Giove , F. Barchetti , F. Podo , and R. Passariello , “High‐Field PET/MRI and MRS: Potential Clinical and Research Applications,” Clinical and Translational Imaging 1, no. 1 (2013): 17–29.

[28] J. Mauler , P. Lohmann , A. A. Maudsley , et al., “Diagnostic Accuracy of MR Spectroscopic Imaging and ^18^F‐FET PET for Identifying Glioma: A Biopsy‐Controlled Hybrid PET/MRI Study,” Journal of Nuclear Medicine 65, no. 1 (2024): 16–21. 37884332 10.2967/jnumed.123.265868

[29] X. Zhang , Y.‐L. E. Chen , R. Lim , C. Huang , I. A. Chebib , and G. El Fakhri , “Synergistic Role of Simultaneous PET/MRI‐MRS in Soft Tissue Sarcoma Metabolism Imaging,” Magnetic Resonance Imaging 34, no. 3 (2016): 276–279. 26523656 10.1016/j.mri.2015.10.027 PMC4761342

[30] C. Ma , P. K. Han , T. Marin , Y. Zhuo , H. A. Shih , and G. E. Fakhri , “Multimodality Molecular Imaging of Brain Tumor Using Simultaneous ^18^F FET‐PET/MRSI. 2024 IEEE Nuclear Science Symposium (NSS), Medical Imaging Conference (MIC) and Room Temperature Semiconductor Detector Conference (RTSD). 1–2,” 2024.

[31] L. Zimmer and A. Luxen , “PET Radiotracers for Molecular Imaging in the Brain: Past, Present and Future,” NeuroImage 61, no. 2 (2012): 363–370. 22222719 10.1016/j.neuroimage.2011.12.037

[32] S. B. Hansen and D. Bender , “Advancement in Production of Radiotracers,” Seminars in Nuclear Medicine 52, no. 3 (2022): 266–275. 34836618 10.1053/j.semnuclmed.2021.10.003

[33] D. P. Soares and M. Law , “Magnetic Resonance Spectroscopy of the Brain: Review of Metabolites and Clinical Applications,” Clinical Radiology 64, no. 1 (2009): 12–21. 19070693 10.1016/j.crad.2008.07.002

[34] G. Oz , J. R. Alger , P. B. Barker , et al., “Clinical Proton MR Spectroscopy in Central Nervous System Disorders,” Radiology 270, no. 3 (2014): 658–679. 24568703 10.1148/radiol.13130531 PMC4263653

[35] P. B. Barker and D. D. M. Lin , “In Vivo Proton MR Spectroscopy of the Human Brain,” Progress in Nuclear Magnetic Resonance Spectroscopy 49, no. 2 (2006): 99–128.

[36] G. Öz , D. K. Deelchand , J. P. Wijnen , et al., “Advanced Single Voxel ^1^H Magnetic Resonance Spectroscopy Techniques in Humans: Experts' Consensus Recommendations,” NMR in Biomedicine 34 (2020): e4236. 10.1002/nbm.4236 PMC7347431 31922301

[37] R. Faghihi , B. Zeinali‐Rafsanjani , M.‐A. Mosleh‐Shirazi , et al., “Magnetic Resonance Spectroscopy and Its Clinical Applications: A Review,” Journal of Medical Imaging and Radiation Sciences 48, no. 3 (2017): 233–253. 31047406 10.1016/j.jmir.2017.06.004

[38] I. Tkác , Z. Starcuk , I. Y. Choi , and R. Gruetter , “In Vivo ^1^H NMR Spectroscopy of Rat Brain at 1 Ms Echo Time,” Magnetic Resonance in Medicine 41, no. 4 (1999): 649–656. 10332839 10.1002/(sici)1522-2594(199904)41:4<649::aid-mrm2>3.0.co;2-g

[39] D. K. Deelchand , A. Berrington , R. Noeske , et al., “Across‐Vendor Standardization of Semi‐LASER for Single‐Voxel MRS at 3T,” NMR in Biomedicine 34, no. 5 (2021): e4218. 31854045 10.1002/nbm.4218 PMC7299834

[40] G. Öz and I. Tkáč , “Short‐Echo, Single‐Shot, Full‐Intensity Proton Magnetic Resonance Spectroscopy for Neurochemical Profiling at 4 T: Validation in the Cerebellum and Brainstem,” Magnetic Resonance in Medicine 65, no. 4 (2011): 901–910. 21413056 10.1002/mrm.22708 PMC3827699

[41] D. W. J. Klomp , A. K. Bitz , A. Heerschap , and T. W. J. Scheenen , “Proton Spectroscopic Imaging of the Human Prostate at 7 T,” NMR in Biomedicine 22, no. 5 (2009): 495–501. 19170072 10.1002/nbm.1360

[42] T. W. J. Scheenen , D. W. J. Klomp , J. P. Wijnen , and A. Heerschap , “Short Echo Time ^1^H‐MRSI of the Human Brain at 3T With Minimal Chemical Shift Displacement Errors Using Adiabatic Refocusing Pulses,” Magnetic Resonance in Medicine 59, no. 1 (2008): 1–6. 17969076 10.1002/mrm.21302

[43] R. Lattanzi , A. K. Grant , J. R. Polimeni , et al., “Performance Evaluation of a 32‐Element Head Array With Respect to the Ultimate Intrinsic SNR,” NMR in Biomedicine 23, no. 2 (2010): 142–151. 19904727 10.1002/nbm.1435 PMC2830315

[44] S. W. Provencher , “Estimation of Metabolite Concentrations From Localized In Vivo Proton NMR Spectra,” Magnetic Resonance in Medicine 30, no. 6 (1993): 672–679. 8139448 10.1002/mrm.1910300604

[45] A. Lin , O. Andronesi , W. Bogner , et al., “Minimum Reporting Standards for In Vivo Magnetic Resonance Spectroscopy (MRSinMRS): Experts' Consensus Recommendations,” NMR in Biomedicine 34, no. 5 (2021): e4484. 33559967 10.1002/nbm.4484 PMC8647919

[46] T. K. Koo and M. Y. Li , “A Guideline of Selecting and Reporting Intraclass Correlation Coefficients for Reliability Research,” Journal of Chiropractic Medicine 15, no. 2 (2016): 155–163. 27330520 10.1016/j.jcm.2016.02.012 PMC4913118

[47] G. Emvalomenos , S. Trajanovska , B. T. T. Pham , et al., “Performance Evaluation of a PET Insert for Preclinical MRI in Stand‐Alone PET and Simultaneous PET–MRI Modes,” EJNMMI Physics 8, no. 1 (2021): 68. 34626239 10.1186/s40658-021-00415-1 PMC8502182

