# Supplementary material for: Comparison of Magnetic Resonance Spectra Acquired With Hybrid PET/MR and Standalone MR Scanners
Source: J Magn Reson Imaging. 2025 Aug 4;62(6):1854–65. doi: 10.1002/jmri.70056 (PMC12604544; doi:10.1002/jmri.70056)
Supplement: Supplementary file 1 — Table S1: Hierarchical linear mixed model comparing in vivo metrics and metabolite concentrations from spectra acquired on hybrid PET/MR pre‐upgrade to post‐upgrade. Table S2: Hierarchical linear mixed model comparing in vitro metrics and metabolite concentrations from spectra acquired on hybrid PET/MR pre‐upgrade to post‐upgrade. [file JMRI-62-1854-s001.docx]

**Comparison of magnetic resonance spectra acquired with hybrid PET/MR and standalone MR scanners**

**Supplementary Information**

**Contents:**

**Supplementary Table 1.** Hierarchical linear mixed model comparing in vivo metrics and metabolite concentrations from spectra acquired on hybrid PET/MR pre-upgrade to post-upgrade.

**Supplementary Table 2.** Hierarchical linear mixed model comparing in vitro metrics and metabolite concentrations from spectra acquired on hybrid PET/MR pre-upgrade to post-upgrade.

**Appendix 1.** MRSinMRS checklist

**Supplementary Table 1.** Hierarchical linear mixed model comparing in vivo metrics and metabolite concentrations from spectra acquired on hybrid PET/MR pre-upgrade to post-upgrade.

| **Metric** | **Upgrade** | **Estimate^a^** | **Standard Error** | **Degrees of Freedom** | **t-statistic** | **p-value** |
| --- | --- | --- | --- | --- | --- | --- |
| SNR | Pre-upgrade | 116.6 | 4.3 | 22.3 | 0.7 | 0.51 |
|  | Post-upgrade | 120.2 | 5.3 | 22.3 |  |  |
| FWHM (Hz) | Pre-upgrade | 7.0 | 0.3 | 90.0 | 1.0 | 0.31 |
|  | Post-upgrade | 7.3 | 0.4 | 90.0 |  |  |
| tNAA/tCr | Pre-upgrade | 1.58 | 0.05 | 21.0 | 0.5 | 0.59 |
|  | Post-upgrade | 1.61 | 0.06 | 21.0 |  |  |
| tCho/tCr | Pre-upgrade | 0.20 | <0.01 | 21.0 | 2.3 | 0.033 |
|  | Post-upgrade | 0.21 | <0.01 | 21.0 |  |  |
| Glu/tCr | Pre-upgrade | 1.18 | 0.03 | 21.0 | 2.8 | 0.012 |
|  | Post-upgrade | 1.29 | 0.04 | 21.0 |  |  |
| Ins/tCr | Pre-upgrade | 0.67 | 0.02 | 21.0 | 1.5 | 0.14 |
|  | Post-upgrade | 0.70 | 0.02 | 21.0 |  |  |

^a^ Estimates reflect mixed model estimates, with pre-upgrade values used as the reference. Standard errors and degrees of freedom were obtained using the Satterthwaite approximation. Pre-upgrade software version = 26.003, Post-upgrade software version = 30.1; FWHM = full width at half maximum; SNR = signal-to-noise ratio; tCr = total creatine + phosphocreatine; tNAA = total *N*-acetylaspartate + *N*-acetyl-aspartyl-glutamate; tCho = total glycerophosphocholine + phosphocholine; Glu = glutamate; Ins = myo-inositol

**Supplementary Table 2.** Hierarchical linear mixed model comparing in vitro metrics and metabolite concentrations from spectra acquired on hybrid PET/MR pre-upgrade to post-upgrade.

| **Metric** | **Upgrade** | **Estimate^a^** | **Standard Error** | **Degrees of Freedom** | **t-statistic** | **p-value** |
| --- | --- | --- | --- | --- | --- | --- |
| SNR | Pre-upgrade | 313.3 | 10.2 | 18.0 | 2.2 | 0.041 |
|  | Post-upgrade | 344.9 | 14.4 | 18.0 |  |  |
| FWHM (Hz) | Pre-upgrade | 2.2 | 0.1 | 18.0 | -0.4 | 0.70 |
|  | Post-upgrade | 2.1 | 0.1 | 18.0 |  |  |
| NAA/Cr | Pre-upgrade | 1.21 | <0.01 | 38.0 | 0.7 | 0.46 |
|  | Post-upgrade | 1.22 | <0.01 | 38.0 |  |  |
| Cho/Cr | Pre-upgrade | 0.31 | <0.01 | 18.0 | 0.1 | 0.95 |
|  | Post-upgrade | 0.31 | <0.01 | 18.0 |  |  |
| Glu/Cr | Pre-upgrade | 0.87 | <0.01 | 18.0 | 10.7 | <0.0001 |
|  | Post-upgrade | 0.94 | <0.01 | 18.0 |  |  |
| Ins/Cr | Pre-upgrade | 0.57 | <0.01 | 18.0 | 11.9 | <0.0001 |
|  | Post-upgrade | 0.59 | <0.01 | 18.0 |  |  |
| Lac/Cr | Pre-upgrade | 0.37 | <0.01 | 18.0 | -0.5 | 0.66 |
|  | Post-upgrade | 0.37 | <0.01 | 18.0 |  |  |

^a^ Estimates reflect mixed model estimates, with pre-upgrade values used as the reference. Standard errors and degrees of freedom were obtained using the Satterthwaite approximation. Pre-upgrade software version = 26.003; Post-upgrade software version = 30.1; FWHM = full width at half maximum; SNR = signal-to-noise ratio; Cr = creatine; NAA = *N*-acetylaspartate; Cho = choline; Glu = glutamate; Ins = myo-inositol; Lac = lactate**Appendix 1. MRSinMRS checklist**

See *Minimum reporting standards for in vivo magnetic resonance spectroscopy (MRSinMRS): experts’ consensus recommendations* as described in:

Lin A, Andronesi O, Bogner W, et al. Minimum reporting standards for in vivo magnetic resonance spectroscopy (MRSinMRS): Experts' consensus recommendations. NMR Biomed 2021;34(5):e4484.

| 1. **Hardware** | |
| --- | --- |
| 1. Field Strength [T] | 3 T |
| 1. Manufacturer | GE, Siemens |
| 1. Model | GE Signa^TM^ PET/MR Siemens MAGNETOM Prisma Fit MR  GE Signa^TM^ Premier MR |
| 1. RF coils | 19 channel head-neck (GE PET/MR)  32 channel head-neck (Siemens MR)  21 channel head-neck (GE MR) |
| 1. Additional hardware | N/A |
| 1. **Acquisition** |  |
| 1. Pulse Sequence | semi-LASER |
| 1. Volume of Interest (VOI) Locations | Posterior cingulate cortex in human brain; isocenter in Braino phantom |
| 1. Nominal VOI size [cm^3^] | 2 x 2 x 2 cm^3^ |
| 1. Repetition time (T _R_), echo time (T _E_) [ms] | T_R_ / T_E_ = 2000/35 ms |
| 1. Total number of excitations or acquisitions per spectrum | 128 in vivo, 64 in vitro |
| 1. **Data analysis methods and outputs** |  |
| 1. Analysis Software | LCModel (version 6.3-*1R*), MATLAB (version 2023a) |
| 1. Processing steps deviating from quoted reference or product | Spectra were analyzed from 4.0 to –1 ppm to include an upfield, noise-only region for analysis |
| 1. Output measure | Metabolite ratios normalized to total creatine, SNR and FWHM calculated from fitted *N*-acetylaspartate (NAA) peak from 1.9 to 2.1 ppm. |
| 1. Quantification references and assumptions, fitting model assumptions | GAMMA-simulated basis set used for fitting consisting of 27 metabolites (alanine, aspartate, methyl creatine, methylene creatine, phosphocreatine, gamma-aminobutyric acid, glucose, glutamine, glutamate, glycerophosphocholine, phosphocholine, glutathione, Ins, Lac, *N*-acetylaspartate, *N*-acetyl-aspartyl-glutamate, scyllo-inositol, taurine, four lipid resonances, and five macromolecular resonances) |
| 1. **Data quality** |  |
| 1. Reported variables | SNR, FWHM, ICC of repeated measures, MSE of metabolite quantification |
| 1. Data exclusion criteria | Excessive movement in scanner in vivo, in vitro FWHM >10 Hz, metabolite CRLBs >20% |
| 1. Quality measures of postprocessing model fitting | CRLBs |
| 1. Sample Spectra | Figure 2 |

semi-LASER = semi-localized by adiabatic selective refocusing; SNR = signal-to-noise ratio; FWHM = full width at half maximum; ICC = intraclass correlation coefficients; MSE = mean squared error; CRLBs = Cramer-Rao lower bounds
